# Optimization of seizure prevention by cannabidiol (CBD)

**DOI:** 10.1515/tnsci-2022-0362

**Published:** 2025-03-28

**Authors:** Bidhan Bhandari, Sahar Emami Naeini, Sholeh Rezaee, Hannah M. Rogers, Hesam Khodadadi, Asamoah Bosomtwi, Mohammad Seyyedi, Neil J. MacKinnon, Krishnan M. Dhandapani, Évila Lopes Salles, David C. Hess, Jack C. Yu, Debra Moore-Hill, Fernando L. Vale, Lei P. Wang, Babak Baban

**Affiliations:** Department of Oral Biology and Diagnostic Sciences, Dental College of Georgia, Augusta University, Augusta, GA, United States of America; Center for Excellence in Research, Scholarship and Innovation (CERSI), Dental College of Georgia, Augusta, Augusta University, Augusta, GA, United States of America; The Graduate School, Augusta University, Augusta, GA, United States of America; Medical College of Georgia, Augusta University, Augusta, GA, United States of America; College of Education, Augusta University, Augusta, GA, United States of America; Department of Neurology, Medical College of Georgia, Augusta University, Augusta, GA, United States of America; Georgia Cancer Center and Department of Biochemistry and Molecular Biology, Medical College of Georgia, Augusta University, Augusta, GA, United States of America; Piedmont Ear, Nose, Throat and Related Allergy, Atlanta, GA, United States of America; School of Public Health, Augusta University, Augusta, GA, United States of America; Department of Neurosurgery, Medical College of Georgia, Augusta University, Augusta, GA, United States of America; Department of Surgery, Medical College of Georgia, Augusta University, Augusta, GA, United States of America

**Keywords:** CBD, epilepsy, seizure, route of administration, inhaler, epidiolex

## Abstract

**Objective:**

Cannabidiol (CBD) is one of the most prominent non-psychotropic cannabinoids with known therapeutic potentials. Based on its anti-seizure efficacy, the first cannabis derived pharmaceutical grade CBD-based medication was approved in the USA in 2018 for the treatment of seizures in patients 2 years and older. Despite the effectiveness in reducing seizures, there remain several major questions on the optimization of CBD therapy for epilepsy such as the optimal dosage, composition, and route of delivery, which are the main objective of this current study.

**Methods:**

We evaluated the antiseizure effects of CBD through different compositions, routes of delivery, and dosages in a pre-clinical model. We used a kainic acid-induced epilepsy model in C57BL/6 mice, treated them with placebo and/or CBD through inhalation, oral, and injection (intraperitoneal) routes. We used CBD broad spectrum (inhaled and intraperitoneal) vs CBD isolate formulations. We employed the Racine scaling system to evaluate the severity of the seizures, flow cytometry for measuring immune biomarkers and neurotrophic factors, and histologic analysis to examine and compare the groups.

**Results:**

Our findings showed that all forms of CBD reduced seizures severity. Among the combination of CBD tested, CBD broad spectrum via inhalation was the most effective in the treatment of epileptic seizures (*p* < 0.05) compared to other forms of CBD treatments.

**Conclusion:**

Our data suggest that route and CBD formulations affect its efficacy in the prevention of epileptic seizures. Inhaled broad spectrum CBD showed a potential superior effect compared to other delivery routes and CBD formulations in the prevention of epileptic seizures, which warrants further research.

## Abbreviations

ASM: anti-seizure medications
BDNF: brain-derived neurotrophic factor
CBD: cannabidiol
CNS: central nervous system
FDA: Food and Drug Administration
IC: immune checkpoint
IL-33: interleukin 33
IL-6: interleukin 6
IP: intraperitoneal
KA: kainic acid
PD1: programmed cell death protein 1
ROA: route of administration
THC: delta-9-tetrahydrocannabinol
TrkB: tropomyosin receptor kinase B

## 1 Introduction

Cannabinoids are closely related naturally occurring compounds found in *Cannabis sativa*. The cannabis plant has been used for its medicinal purposes since ancient times all over the world [1]. However, it was only in the last half century and particularly for last two decades that the public and scientific interests have mounted significantly, triggering intense and extensive research in systematic medical characterization of cannabinoids [1]. The cannabis plant produces over 500 compounds, but only 100 of them are classified as cannabinoids, and two of them, delta-9-tetrahydrocannabinol (THC) and Cannabidiol (CBD) being the most known and investigated cannabinoids for their medical as well as recreational purposes [2,3]. While THC is recognized and well-known for its psychoactive features, CBD is non-psychotropic, with potential anti-inflammatory effects, and a high therapeutic index [4]. Recent work by our laboratory and others suggests beneficial effects of CBD alone or in combination with other cannabinoids in the treatment of several pathologic conditions including neurologic diseases as well as malignancies [5,6,7,8]. Importantly, several reports have suggested that CBD could be used as an anti-convulsive and well-tolerated agent with beneficial effects in the treatment of seizures [9,10]. It was in 2018 when the US Food and Drug Administration (FDA) approved the first pharmaceutical dosage form of CBD for treating patients 2 years and older with Dravet syndrome or Lennox-Gastaut syndrome, two of the various, rare epileptic disorders classified as epileptic encephalopathies [10,11]. The treatment later received European Commission approval to be available for patients across Europe [12].

Epilepsy is a common and chronic neurologic condition characterized by recurrent and wanton seizures [13]. Affecting over 50 million individuals at all ages globally, epilepsy remains one of the most challenging disorders with direct, indirect, and intangible costs to individuals, healthcare system, and the economy overall, such as worker productivity [13,14,15]. Even with the progress made in developing anti-seizure medications, there remains a significant need for new treatments. Current drugs often face challenges related to their effectiveness, side effects, tolerability, and variability in individual response. Research is actively exploring new mechanisms of action, personalized treatment strategies, and medications with improved pharmacokinetic properties. These efforts aim to enhance patient outcomes, particularly for those with drug-resistant epilepsy, coexisting medical conditions, or those in pediatric and geriatric age groups. The introduction of these new therapies could provide more effective, safer, and better-tolerated options, significantly improving the quality of life for people with epilepsy [16,17,18,19].

The immune system, particularly cytokines and immune checkpoints (ICs), plays a crucial role in the pathophysiology of seizures and epilepsy [20,21,22]. Cytokines, as key modulators of inflammation, can affect neuronal excitability and synaptic plasticity, contributing to both the onset and progression of seizures. Dysregulated immune responses, including the activation of ICs like PD-1, may influence neuroinflammation and neuronal network dysfunction, further exacerbating seizure activity and promoting epilepsy. Monitoring cytokine levels and IC activity is important because these molecules can serve as biomarkers for the inflammatory processes underlying seizures, and their modulation may offer new therapeutic strategies. By measuring cytokines and ICs, researchers and clinicians can better understand how immune responses contribute to seizure control and epilepsy progression, paving the way for targeted interventions aimed at restoring immune balance and improving patient outcomes [20,21,22].

Although some research has explored the use of inhaled CBD for conditions like anxiety and pain, its application in epilepsy treatment is not well-studied. Oral CBD, especially in the form of Epidiolex, which is FDA-approved for severe pediatric epilepsy, is the most common and thoroughly researched method [23,24,25,26,27,28,29]. Several studies have demonstrated the potential of CBD as an anticonvulsant agent in preclinical rat models of epilepsy, particularly those using the LiCl-pilocarpine method [30]. While inhaled CBD might provide a quicker onset of effects, concerns about lung health and the absence of comprehensive clinical trials currently prevent it from being a standard epilepsy treatment [31]. There is potential for future research in this area, but for now, oral CBD remains the preferred option for managing epilepsy.

In this study, we explored the potential of inhaled CBD as a prophylactic treatment for epileptic seizures in a pre-clinical mouse model. The results were highly encouraging, suggesting that the inhaled CBD preparation may offer effective seizure prevention and could demonstrate superior effectiveness compared to other CBD-based formulations in this context. However, as this study was conducted in a mouse model, further research is needed to assess the therapeutic potential of this approach in humans.

## 2 Materials and methods

### 2.1 Animals

Male C57BL/6 mice purchased from Jackson Laboratories, USA were used in these experiments. Mice were housed as a group and handled according to the National Institute of Health (NIH) guide for the Care and Use of Laboratory Animals. All experiments were conducted under the approval of the Augusta University Animal Care and Use Committee (Protocol # 2011-0062).

### 2.2 Experimental groups and prophylactic treatments with variable delivery routes

**Figure 1:**
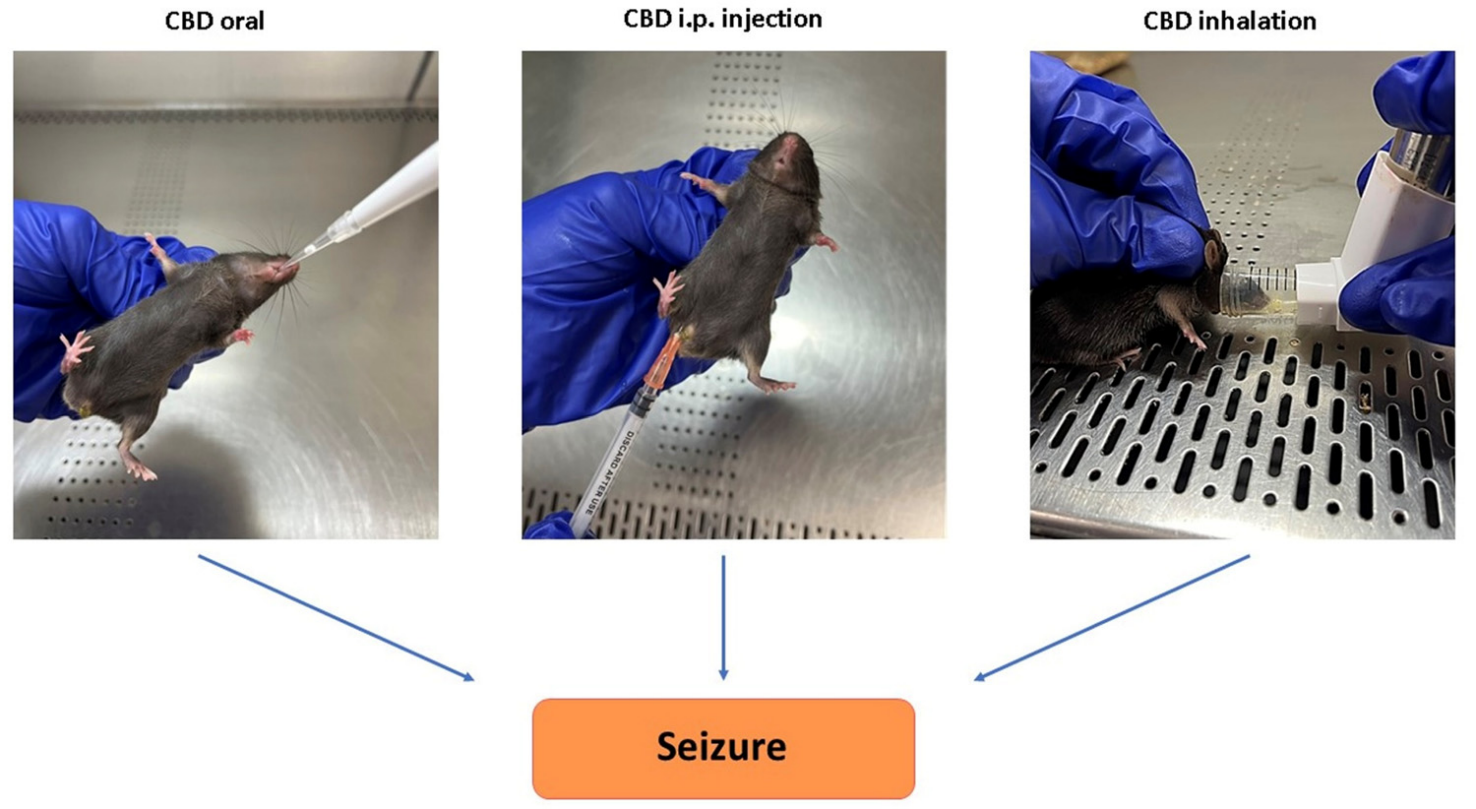
Methods of CBD delivery. Left: Oral administration, simulating clinical CBD-based treatments (e.g., Epidiolex) for epilepsy. Center: Intraperitoneal (IP) injection of CBD. Right: Modified inhaler for CBD delivery via inhalation.

Mice were divided into six groups (*n* = five/group, total of five independent cohorts). The “CBD” group which was treated prophylactically with inhalant CBD (8.5 mg per mouse) using inhaled CBD in three different doses of CBD concentration of 100, 10, and 1% (TM Global Bioscience, USA). These concentrations correspond to different dilutions of the full-strength CBD dose (8.5 mg per mouse). The 100% concentration provides the full 8.5 mg of CBD per mouse, the 10% concentration delivers 10% of the full dose, which is 0.85 mg per mouse, and the 1% concentration offers 1% of the full dose, equaling 0.085 mg of CBD per mouse. The formulation of experimental inhalers was a broad-spectrum CBD containing natural compounds and cannabinoids (except THC). The placebo was administered through inhalers too. However, for the placebo, the whole CBD content was replaced by hemp seed oil, containing no cannabinoids. As depicted in Figure 1, the inhaler was modified by adding an extra nozzle piece to adjust to the mouse model and to further control the intake of CBD. The fourth group, received CBD (broad-spectrum) prophylactically through intra-peritoneal (IP) route (10 mg/kg) while the fifth group (Placebo) received inhaled placebo. The sixth group was treated with CBD isolate through oral gavage (Epidiolex, NDC70127-100-60, Cardinal Health). The CBD isolate formulation contained CBD only with no other cannabinoids and compounds, which makes it differ from our inhaled formulation. After 30 min of CBD/Placebo treatments, the epileptic seizures were induced in all mice using kainic acid (KA) as described hereinafter. It is important to note that the pharmacological comparability of different CBD application routes and concentrations is primarily determined by the total amount of CBD delivered to the subjects. While the method of administration (inhalation, oral, IP injection) and the concentration of CBD (100, 10, 1%) may differ, the key factor for pharmacological comparability is the total dose of CBD administered. Therefore, ensuring that the total CBD dose is consistent across the different application routes and concentrations is crucial for accurately assessing their pharmacological equivalence.

### 2.3 Induction of epileptic seizures: KA infusion

KA (Fisher Scientific) was used to induce epileptic seizures as described previously [32]. Briefly, KA was dissolved in phosphate buffered saline (pH 7.4) and delivered through IP injection (20 mg/kg). The Racine scoring system was used to assess the behavior and severity of the induced epileptic seizures.

### 2.4 Racine scoring system

The Racine scoring system was used to assess the degree of seizure in our experimental epilepsy model. The scoring was based on the standard Racine scale as described previously [33,34]. Briefly, the level of seizure severity was measured with the following stages: (0) no abnormality; (1) Mouth and facial movements; (2) Head nodding; (3) Forelimb clonus; (4) Rearing; and (5) Rearing and falling. While the scoring was performed and monitored in real time, the whole process was video recorded for further and future analysis if needed.

### 2.5 Analytical flow cytometry

To set a pattern of potential predictive, diagnostic, and prognostic biomarkers during the course of study, whole blood and brain samples were collected, processed and analyzed using flow cytometry. Both brain tissues and whole blood were analyzed for immune-inflammatory biomarkers including programmed cell death protein 1 (PD1, an inhibitory receptor), interleukin 33, interleukin 6 (IL-33/IL-6, alarmin/cytokine), and level of brain-derived neurotrophic factor (BDNF).

### 2.6 Preparative flow cytometry

For cell-surface marker of PD-1, immunophenotyping was carried out as described previously (Khodadadi et al. [8]). Briefly, for brain samples, tissues were sieved through a cell strainer (BD Biosciences), followed by centrifugation (252 g, 5 min, 4°C) to prepare single-cell suspensions. Next all samples (whole blood and cell suspension from brain) were incubated with conjugated antibody against PD-1, followed by fixation and permeabilization using fix/perm concentrate (eBioScience). Next samples were incubated with antibodies for intracellular staining of IL-33, IL-6, and BDNF (all antibodies were purchased from Biolegend unless otherwise noted). Cells were then run through a NovoCyte Quanteon flow cytometer. Cells were gated based on forward and side scatter properties and on marker combinations to select viable cells of interest. Single stains were used to set compensation, and isotype controls were used to determine the level of nonspecific binding. Analysis was performed using FlowJo (version 11.0) analytical software. Cells expressing a specific marker were reported as a percentage of the number of gated events. A population was considered positive for a specific marker if the population exceeded a 2% isotypic control threshold.

### 2.7 Statistics

Given the non-continuous status values of Racine scores, the Kruskal-Wallis H Test was employed across all experimental groups (Placebo, Inh CBD, Oral CBD, and Inj CBD) to determine and perform statistical analysis. *Post hoc* pairwise comparisons were conducted by Mann-Whitney U tests *(Wilcoxon Test)* using GraphPad Prism 9. Sta. For the flow cytometry analysis, the one-way ANOVA was used followed by Tukey’s test.


**Ethical approval:** The research related to animals use has been complied with all the relevant national regulations and institutional policies for the care and use of animals.

## 3 Results

### 3.1 Prophylactic treatment with inhaled CBD was able to effectively mitigate severity of KA-induced acute epileptic seizures

**Figure 2:**
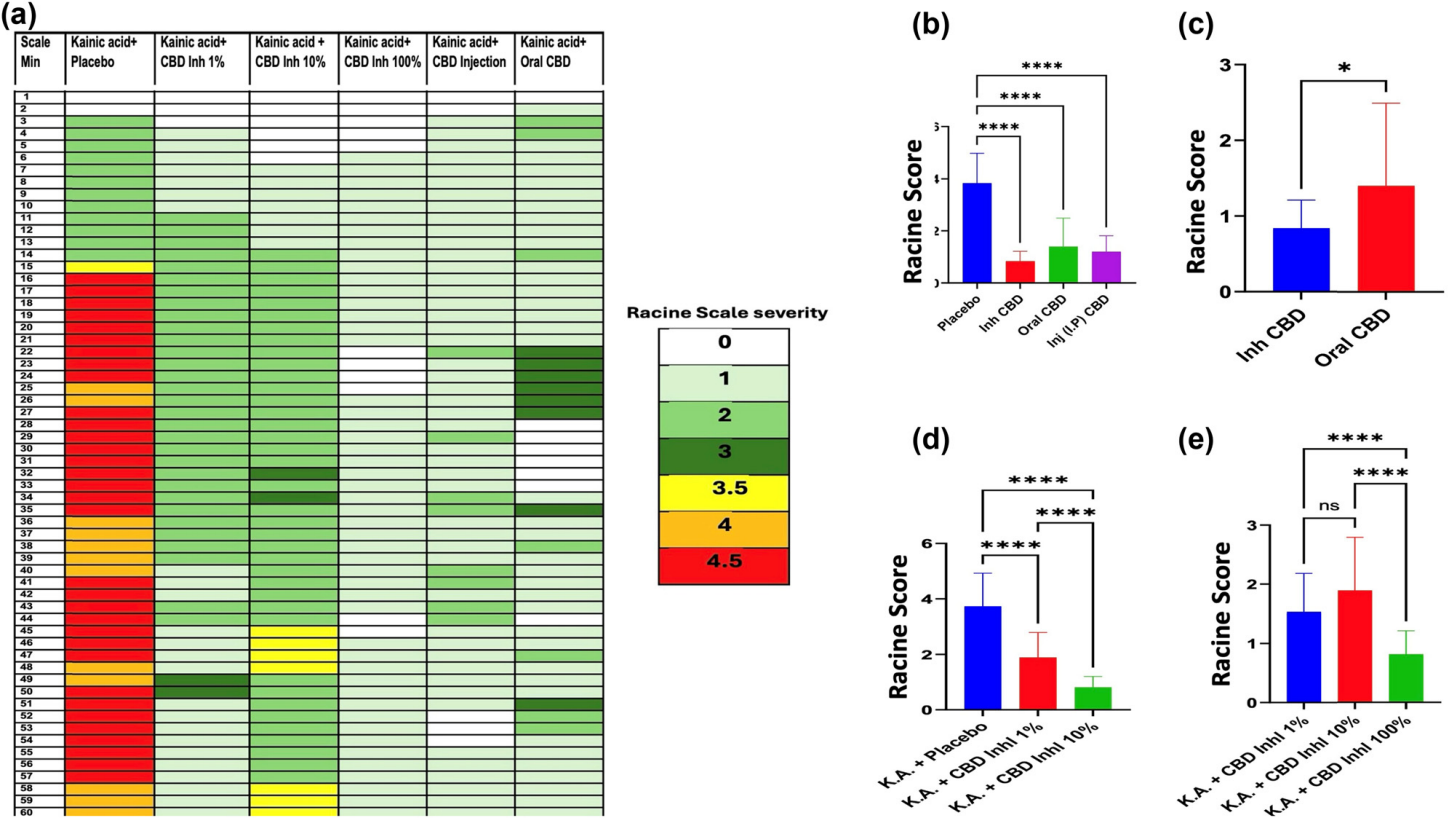
Prophylactic treatment with inhalant CBD reduced severity of KA-induced epileptic seizures. Seizures were induced using 20 mg/kg KA through IP injection to mice. Mice were pre-treated with placebo or one of the variable formulations of CBD including inhaled, oral or IP injection 30 min prior to seizure induction. (a) Seizure severity was video recorded, scored based on Racine scale ranging from stage 0 to 5, with 0 indicating no abnormality; 1, Mouth and facial movements; (2) Head nodding; (3) Forelimb clonus; (4) Rearing; (5) Rearing and falling. (b) Quantified histogram of Racine scoring system: The statistical analysis of Racine scores across the four groups (Placebo, lnh CBD, Oral CBD, and lnj CBD) revealed significant differences, as determined by the Kruskal–Wallis H Test (statistic = 56.20, *p* < 0.0001). *Post hoc* pairwise Dunn multiple comparisons indicated that the Placebo group had significantly higher Racine scores compared to lnh CBD (adjusted *p* < 0.001), Oral CBD (adjusted *p* < 0.001), and lnj IP CBD (adjusted *p* < 0.001). (c) The comparison between the Inhaled CBD (lnh CBD) and Oral CBD (Oral CBD) groups using the Mann-Whitney *U* test (Wilcoxon Test) revealed a statistically significant difference in Racine scores (*U* = 226.5, *p* = 0.029). (d) The statistical analysis of Racine scores across the three groups including placebo vs 1% (0.085 mg/mouse) and 10% (0.85 mg/mouse) of CBD revealed significant differences, as determined by the Kruskal–Wallis H Test (statistic = 56.20, *p* < 0.0001). *Post hoc* pairwise Dunn multiple comparisons indicated that the Placebo group had significantly higher Racine scores compared to 1% inhaled CBD (adjusted *p* < 0.0001), and 10% inhaled CBD (adjusted *p* < 0.0001). (e) The statistical analysis of Racine scores across the three groups including different concentrations of CBD (1, 10, and 100%, where 100% is full strength of 8.5 mg/mouse) revealed significant differences, as determined by the Kruskal–Wallis H Test (statistic = 56.20, *p* < 0.0001). *Post hoc* pairwise Dunn multiple comparisons indicated that the 10% inhaled CBD had significantly higher Racine scores compared to 1% and full strength lnh CBD (adjusted *p* < 0.0001).

Treatment of mice with the inhalant CBD in a prophylactic fashion (30 min prior to seizure induction) reduced the severity of seizures significantly compared to placebo treatment. The seizure severity was measured using the Racine seizure scale from 0–5 as described in methods and shown in Figure 2a. While mice treated with three different concentration (100, 10, and 1%) of inhaled CBD were scored between 1 and 2, the placebo treated mice were scored at 4.5 with full spectrum of severe seizure (Figure 2a–c, *p* < 0.001). Among all three concentrations of CBD, the full strength CBD showed the most effective outcomes with significant differences (Figure 2d and e, *p* < 0.0001).

### 3.2 Efficacy of CBD in the treatment of epileptic seizures was route dependent

As shown in Figure 2b, all three forms of CBD used in this study were able to reduce severity of the seizures. However, the inhalation of CBD was the most effective route of delivery in preventing development of symptoms and reducing severity of seizure with a significant difference compared to oral route or IP injection (*p* < 0.029). Interestingly, the IP route of CBD was more effective in mitigating the seizure severity compared to oral delivery (Figure 2b–e).

### 3.3 CBD treatment alleviated the symptoms and reduced severity of epileptic seizures by regulating the expression of neuro-immunologic factors in a route dependent fashion

#### 3.3.1 Regulation of alarmin and immune mediator

**Figure 3:**
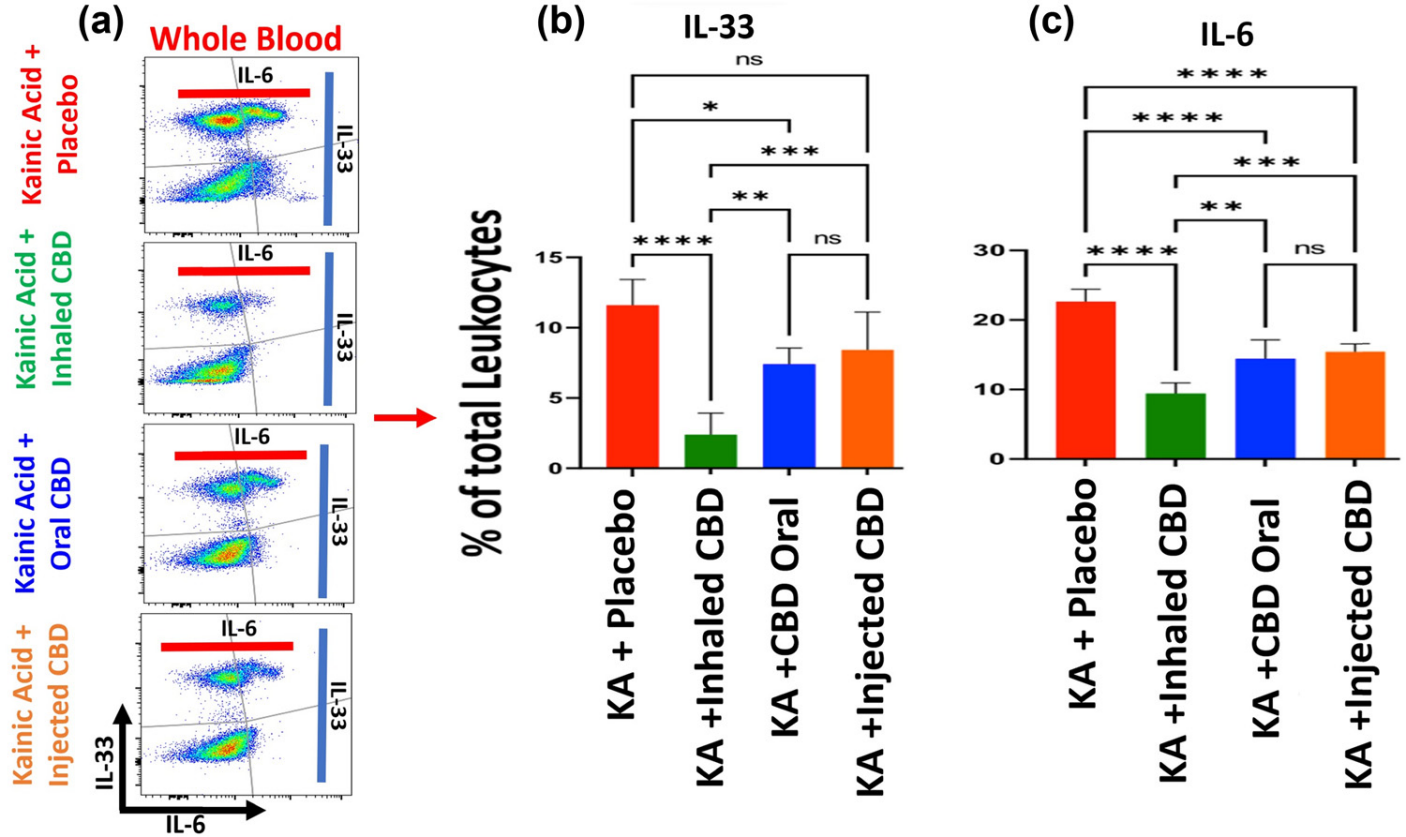
CBD decreased circulating alarmin and pro-inflammatory cytokines in peripheral blood in KA-induced epilepsy. (a) Flow cytometry of whole blood two­dimensional dot plots of IL-33 expressing cells vs IL-6 positive cells in peripheral blood. (b) Quantified bar graphs of flow cytometry analysis of blood demonstrated a significant reduction in IL-33 level in blood during epilepsy when pre-treated with inhalant CBD, highest reduction (****p ≤ 0.0001, *n* = five/group) followed by oral form (*p ≤ 0.05, *n* = five/group). Injected form of CBD delivery, IP, did not result in any significant reduction in IL-33 in peripheral blood (ns). (c) Quantified bar graphs of flow cytometry analysis of blood showed a significant reduction in IL-6 in animals during KA-induced epilepsy pre-treated with all forms of CBD compared to placebo (****p ≤ 0.0001, *n* = five/group). Flow cytometry dot plots are representatives of five animals per experimental group.

**Figure 4:**
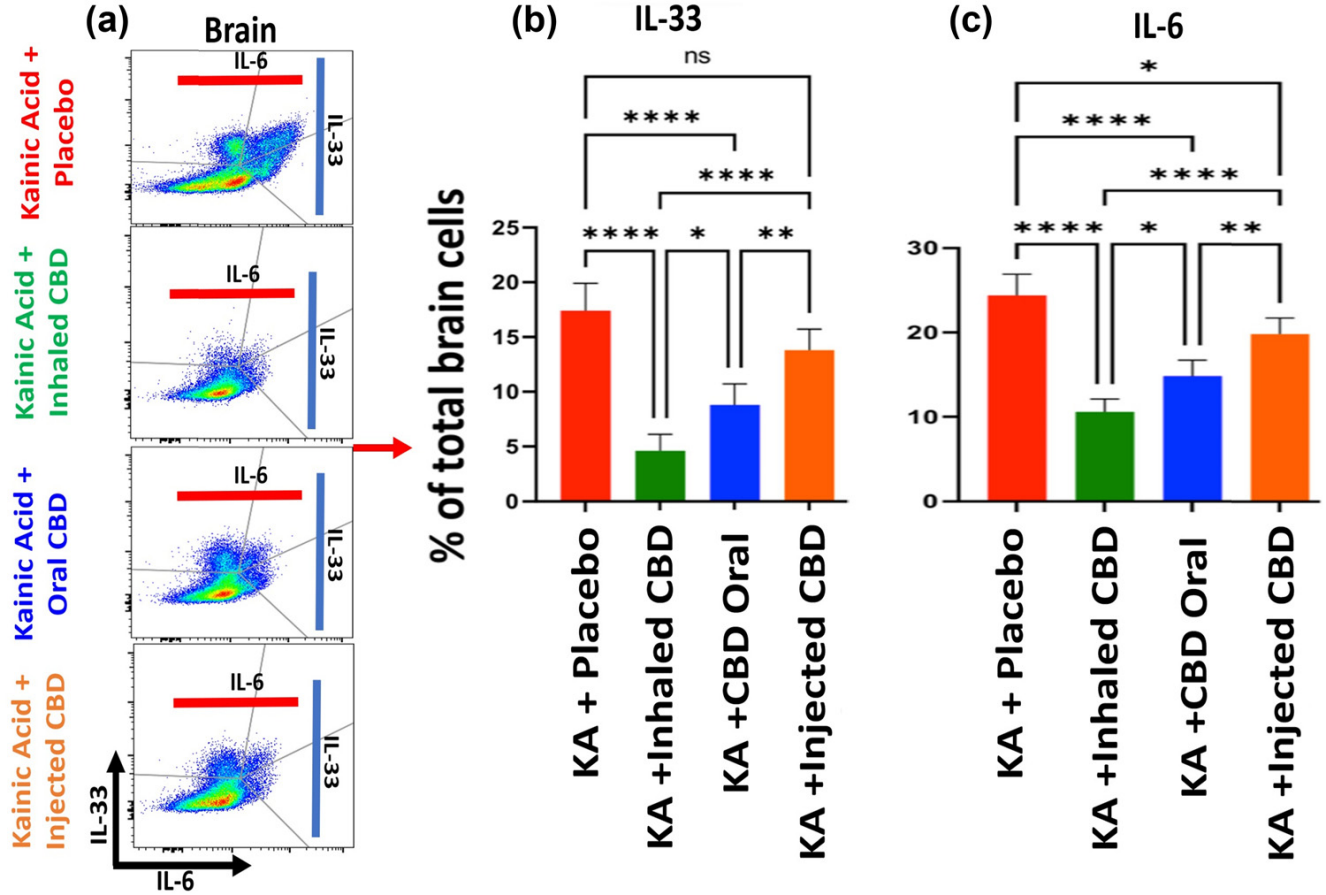
CBD decreased brain alarmin and pro-inflammatory cytokines in KA­ induced epilepsy. (a) Brain flow cytometry two-dimensional dot plots of IL-33 expressing cells vs IL-6 positive cells in brain. (b) Quantified bargraphs of flow cytometry analysis of brain demonstrated a significant reduction in IL-33 level in brain during epilepsy when pre-treated with inhalant CBD or with oral form (****p ≤ 0.0001, *n* = five/group). Similar to blood, IP form of CBD delivery did not result in significant reduction in IL-33 in brain (ns). (c) Quantified bargraphs of flow cytometry analysis of brain showed a significant reduction in IL-6 in animals during KA-induced epilepsy pre-treated with all forms of CBD. CBD delivery through inhalation and oral route had identical impact on regulation of IL-6 expression (****p ≤ 0.0001, *n* = five/group) while the impact of CBD IP injection on brain IL-6 was still significant; however, with lower level of differences compared to placebo group (*p ≤ 0.05, *n* = five/group). Flow cytometry dot plots are representatives of five animals per experimental group.

Flow cytometry analysis demonstrated that CBD treatment was able to modulate inflammatory responses in both peripheral blood as well as in central nervous system (CNS). CBD decreased the expression level of pro-inflammatory cytokines IL-33 and IL-6 in a route dependent manner (Figures 3 and 4a–c). CBD inhalation showed the most significant measured differences in IL-33 and IL-6 compared to the placebo group (****p ≤ 0.0001). While oral form of CBD reduced both IL-33 and IL-6 with less significance than inhaled CBD, the IP injection of CBD lowered the expression of IL-33 and IL-6 with no significant and/or with minimal differences compared to placebo group. Such important and novel findings indicated the significance of route of administration and the outcomes.

#### 3.3.2 Alteration of BDNF

**Figure 5:**
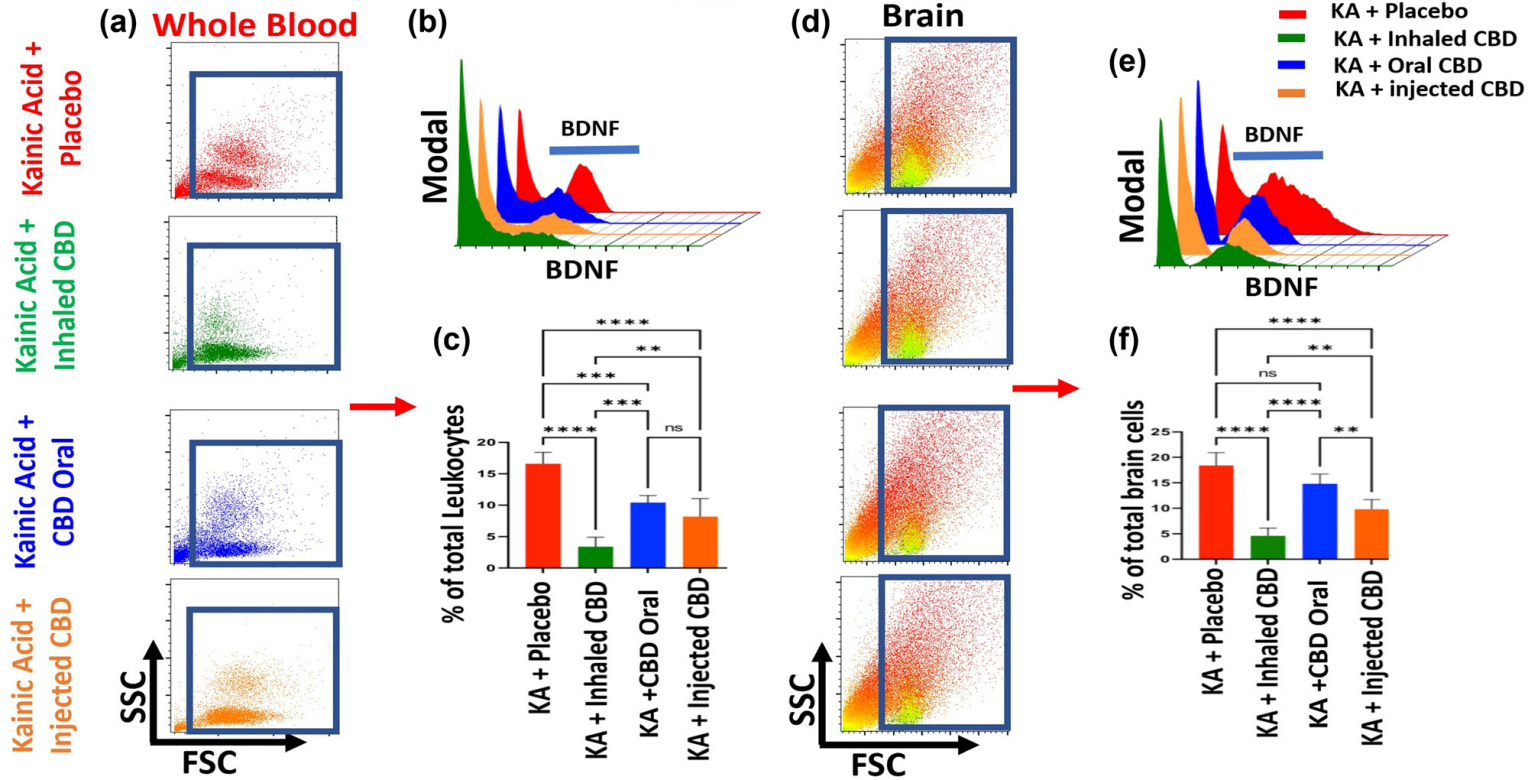
Inhalant CBD reduced BDNF significantly in KA-induced epilepsy. Flow cytometry analysis of peripheral blood demonstrated a significant reduction in BDNF level of mice during epilepsy when pre-treated with CBD. The most reduction in circulating BDNF of blood was when mice pre-treated with either inhalant or IP CBD (****p ≤ 0.0001, *n* = five/group), while oral form resulted in less reduction (***p ≤ 0.001, *n* = five/group). (a) Forward scatter vs side scatter dot plot of whole blood (FSC/SSC), (b) three-dimensional comparative histograms of BDNF in blood of all experimental groups, and (c) quantified bar graph of blood's level of BDNF in all experimental groups. For brain tissues, while flow cytometry analysis showed the most significant reduction in brain's BDNF in mice pre-treated with either inhalant or injected CBD (****p ≤ 0.0001, *n* = five/group); however, the oral form of CBD did not result in any significant reduction in brain's BDNF compared to placebo group (ns). (d) FSC/SSC dot plots of brain tissues, (e) three-dimensional comparative histograms of BDNF in brain tissues of all experimental groups, and (f) quantified bar graph of brain's level of BDNF in all experimental groups. Flow cytometry dot plots are representatives of five animals per experimental group.

Flow cytometry analysis demonstrated that CBD treatment was able to reduce the expression level of BDNF in both peripheral blood and brain, with the most significant decrease in the group treated with inhaled CBD, followed by IP route. Although, the oral administration of CBD showed reduction in the level of BDNF, such decrease was not significant and/or minimal compared to placebo group (Figure 5).

#### 3.3.3 Regulation of ICs

**Figure 6:**
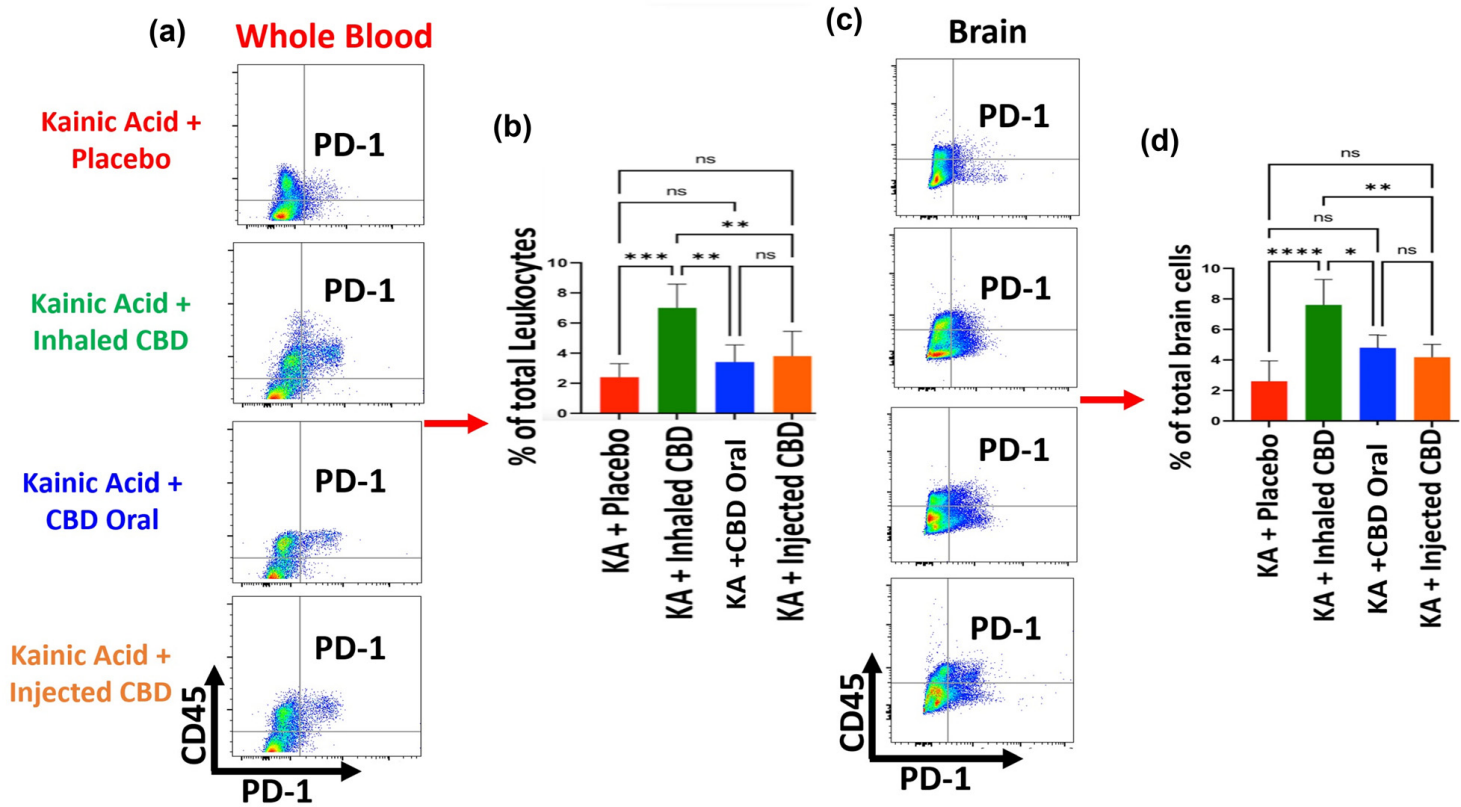
Inhalant CBD increased IC, PD-1, in KA-induced epilepsy. Flow cytometry analysis of peripheral blood demonstrated a significant elevation in circulating PD-1 level of mice in blood during epilepsy when pre-treated with inhalant CBD (***p ≤ 0.001, *n* = five/group). Oral form or injected form of CBD (IP) did not result in any significant increase in PD-1 (ns). (a) Whole blood flow cytometry two­dimensional dot plots of leukocytes (CD45+ cells) vs PD-1 expressing cells in the whole blood, (b) quantified bar graph of blood's level of PD-1 in all experimental groups. For brain tissues, while flow cytometry analysis showed a significant increase in brain's PD-1 in mice pre-treated with inhalant CBD (****p ≤ 0.0001, *n* = five/group), the IP, or oral form of CBD, did not result in any significant increases in brain's PD-1 compared to placebo group (ns). (c) Brain tissue's two-dimensional flow cytometry dot plots of microglia/macrophages (CD45+ cells) vs PD-1 expressing cells in brain tissues, and (d) quantified bar graph of brain's level of PD-1 in all experimental groups. Flow cytometry dot plots are representatives of five animals per experimental group.

Flow cytometry analysis demonstrated that CBD was able to upregulate the expression of IC and PD-1 in both peripheral blood and in the brain (Figure 6a–c). Although, CBD in all forms of administration regulated the PD-1, it was only inhalant CBD that resulted in a significant difference in PD-1 expression (***p ≤ 0.001) compared to placebo group in both peripheral blood as well as in the brain. Both oral or injected form of CBD administration did not result in a significant difference in the expression level of PD-1 in blood and brain compared to the placebo group.

## 4 Discussion

Our findings demonstrate that CBD pre-treatment reduced the severity of the epileptic seizures in an experimental model. Most importantly, our data showed, for the first time, that CBD efficacy could be route- and type-dependent, with inhaled CBD broad spectrum resulting in the most substantial reduction in the severity of the seizures and limiting the symptoms compared to other routes of administration (ROA) and type of CBD. Given the crucial role of ROA, such novel findings could be a major determinant in the therapeutic processes to ensure that medications are delivered to the targeted sites accurately and effectively [35,36]. ROA can influence drug bioavailability, affecting local and systemic absorption, contributing to the speed of action, latency, and intensity of medications [35,36]. Importantly, a desirable ROA should be patient friendly with minimal side effects in a non-invasive fashion [35,36]. Although, several reports have described advantages and disadvantages of common ROAs for CBD (oral, sublingual, topical, inhalant, intranasal, rectal, and parenteral) individually [37,38], to date, no conclusive studies have investigated the features and outcomes of ROAs in a comparative fashion specifically regarding epileptic seizures with CBD treatment. Our findings in this study suggest that inhaled CBD may offer a potential novel approach for treating epileptic seizures. Additionally, the data indicate that inhaled CBD could show some advantages over other ROAs, such as oral and intraperitoneal injection, although the differences observed were statistically significant and warrant further investigation to confirm these trends. The first-pass effect is a key factor that diminishes the efficacy of a drug, particularly when administered orally. This phenomenon causes a significant reduction in the drug’s concentration and effectiveness before it reaches its intended target. In contrast, the inhalation route not only speeds up the delivery and improves accuracy but also enhances efficacy by bypassing the first-pass effect, allowing for faster and more potent therapeutic effects. Furthermore, inhalers use a lower dose compared to the oral route in safer and more cost-effective fashion. Other advantages of inhaled therapies compared to other ROAs include higher efficacy, less invasive, faster absorption with higher bioavailability and fewer systemic side effects.

Further, our data showed, for the first time, that CBD treatment regulated immunologic responses to seizures by lowering proinflammatory cytokines (IL-33 and IL-6) production and enhancing the level of IC protein of PD-1 within the CNS as well as systemically in the peripheral blood. Increasing evidence points to inflammation and oxidative stress as two major contributing factors in the formation, permanence, and recurrence of seizures [39,40]. Cytokines and their receptors are major mediators and modulators of inflammatory responses within the CNS and peripheral tissues. In fact, the impact of cytokines’ functions within the CNS goes beyond immunologic modulation, affecting neurons acting as neuromodulators as described by several studies [41]. IL-33 is a pleiotropic cytokine and a member of the IL-1 family, which is released as an alarm signal (alarmin), as part of pattern-recognition system: Damage-Associated Molecular Pattern, from apoptosis and necrosis when cells are under stress conditions [42]. Therefore, it is logical to target IL-33 as a neuro-immunotherapeutic modality in the treatment of epileptic seizures [43]. Nevertheless, several studies have suggested a dichotomic function of IL-33 during neuro-inflammatory responses [44,45]. Such dual action means that the impact of IL-33 could be affected by several factors including, not limited to, epigenetic and micro-environmental variables. While such binary action of IL-33 magnifies its central role in the inflammatory responses within the CNS, it also accentuates the significance of CBD as a key regulator, not a suppressor, of IL-33 in optimal and timely modulation of IL-33. A recent report from our group showed a role for CBD-induced regulation of IL-33 in a murine model of Alzheimer’s disease [8]. Understanding the mechanisms by which CBD influences the IL-33 shift from a damaging pro-inflammatory alarmin to a protective cytokine with therapeutic values requires further research and elucidation. IL-6 is also a pro-inflammatory cytokine with convulsive nature contributing to the progression and severity of epileptic seizures [46,47]. Several studies have shown increased level of IL-6 during inflammation within the CNS domain significantly higher than homeostasis and normal condition [47]. Our findings here and in previous studies, have shown that CBD can regulate IL-6 effectively in different pathologic conditions [48]. Such potential could be further investigated as an innovative and effective therapeutic modality in the treatment of a wide spectrum of inflammatory diseases such as epilepsy and its complications. That being said, it is important to note that inflammation is a consequence of the entire epileptic cascade. While it can certainly serve as a useful measure, it should not be regarded as the sole causative factor in the interaction between CBD and the various factors involved in the induction and progression of seizures.

One of the major immunologic molecules in regulation of cytokines and inflammatory mediators is ICs. ICs are gatekeepers of immune responses, restoring and maintaining the immune balance through modulation of innate and adaptive effectors of immune system [49]. PD-1 is considered as a major IC molecule expressed mainly on T-cells as well as several other types of immune cells including B cells, NK cells, macrophages, and dendritic cells [50]. The role of PD-1 in malignancies and auto-immune diseases is well established [51]. Increasing evidence supports the notion that PD-1 can influence the maintenance and re-establishment of homeostasis in CNS during health and in diseases [52]. However, the role of PD-1 in epilepsy has not been fully understood [53]. Few studies have reported the effects of PD-1 in epilepsy or proposed the use of PD-1 as a biomarker in the early diagnosis as well as a therapeutic target in the treatment of epilepsy and seizure prevention [53]. Our findings support the notion of the role of PD-1 in epilepsy and seizure severity. In addition, based on our novel findings, we are further proposing that CBD has the potential to control and ameliorate seizure severity through regulation of PD-1. Inhalant CBD could be a non-invasive immuno-therapeutic modality to contain excessive inflammation by up-regulation of ICs, restoring the immune balance and homeostasis. Such potential could have implications in several other neurologic and inflammatory disorders.

Furthermore, our results demonstrate that induction of epilepsy enhanced the level of BDNF systemically in blood and locally within brain tissues. This was an expected observation since several studies have already demonstrated the link between BDNF and epilepsy [54]. However, the novel findings in this study showed the potential of CBD in the regulation of BDNF in our model of seizure induction. There could be several hypothetical theories to explain the link between CBD and BDNF. It is already reported that it could be a potential connection between cannabinoids and a receptor for BDNF, Tropomyosin receptor kinase B (TrkB) [55]. Therefore, it is possible that CBD may affect the BDNF/TrKB signaling and influence the severity of epileptic seizures, which necessitates further investigation. BDNF expression is driven by activity dependent promoters (such as promoter IV) that respond to neuronal activity and several steady promoters (promoter I, III, and IX in mice) that deliver low and often cells type specific baseline expression. BDNF levels rise transiently following seizures due to activity-dependent transcription from promoter IV. Promoter IV is activated by stimuli like synaptic activity, calcium influx, or action potential firing via transcription factors such as CREB (cAMP response element-binding protein), hence the immediate post epilepsy BDNF levels reflect the degree of seizure-induced neuronal activity, which in this case could serve as a proxy or indicator for epilepsy severity or strength in some cases. It is important to note that BDNF plays a complex and multifaceted role in epilepsy. On one hand, by activating the PI3K-Akt pathway and promoting the expression of anti-apoptotic proteins (such as Bcl-2) while reducing pro-apoptotic factors (like Bax), BDNF supports neuronal survival [56,57,58,59,60]. On the other hand, through the activation of TrkB receptors, BDNF enhances synaptic plasticity, strengthening synaptic connections between neurons, which makes them more excitable and, consequently, more susceptible to excitotoxicity. Through these mechanisms, BDNF can contribute to the development of epilepsy (epileptogenesis) and influence seizure activity [61,62].

Additionally, several reports suggest that different CBD formulations, such as isolate, broad-spectrum, and full-spectrum, may have distinct implications and effects. Broad-spectrum CBD, in particular, offers notable advantages over CBD isolate and full-spectrum CBD due to its unique composition. Unlike isolate, which contains only pure CBD, broad-spectrum CBD retains a variety of other cannabinoids, terpenes, and compounds from the cannabis plant, excluding THC. This composition allows for the “entourage effect,” where the combined action of these compounds enhances the therapeutic potential of CBD [63]. Compared to full-spectrum CBD, broad-spectrum formulations provide the benefits of multiple cannabinoids while reducing the risk of THC-related side effects, making them a safer choice for individuals seeking the synergistic effects of plant compounds without the psychoactive effects of THC. However, further research is needed to fully validate these findings. That being said, it is important to note that while several studies have highlighted the potential therapeutic benefits of inhaled CBD for various conditions [64], the long-term effects of inhaling CBD are still poorly understood, and research on its impact on lung health remains limited. Additionally, concerns have been raised regarding the safety of certain carrier liquids or additives used in CBD products, such as propylene glycol and vitamin E acetate, which have been associated with lung injuries. Individuals with pre-existing respiratory conditions may be at an increased risk, making it crucial to closely monitor for any adverse reactions [65,66].

In conclusion, while CBD has already been shown to have beneficial effects on epileptic seizures, the efficacy of current CBD-based treatments needs optimization by elucidation of mechanism of action, ROA, and formulation. Our findings suggest that ROA and CBD formulation play crucial roles in the efficacy of treatment. CBD delivery through inhalation appears to be more effective in reducing the seizure severity in a non-invasive fashion compared to other existing CBD-based medications for epileptic seizures. These findings are significant and offer a compelling case for further exploration of the ROA and formulation of CBD as a potential treatment for epileptic seizures. However, it is important to acknowledge that these results are based on animal models in a preclinical setting. Translating these findings to human use will require extensive additional research, particularly to address factors such as dosing, efficacy, bioavailability, patient adherence, and safety. While these results provide a strong foundation for future studies, further investigation is essential to gain more applicable insights and refine therapeutic strategies for human application. In addition, this study was specifically designed to evaluate the preventive effects of inhaled CBD, meaning that its ability to prevent seizures before their onset was the primary focus. While these results are promising, they do not address the efficacy of inhaled CBD once seizures have already been induced. Therefore, further studies are required to explore its therapeutic potential in a post-induction context, where CBD could be administered after seizures are triggered to determine whether it can effectively reduce seizure duration, frequency, or severity.
